# Supplementary material for: Perinatal Mother-to-Child Chikungunya Virus Infection: Screening of Cognitive and Learning Difficulties in a Follow-Up Study of the Chimere Cohort on Reunion Island
Source: Viruses. 2025 May 14;17(5):704. doi: 10.3390/v17050704 (PMC12116154; doi:10.3390/v17050704)
Supplement: Supplementary file 1 [file viruses-17-00704-s001.zip › Sarton R et al, Viruses supporting information.pdf]

viruses MDPI

Supporting information

Article

Perinatal mother-to-child chikungunya virus infection : screening of cognitive and learning difficulties in a follow-up study of the CHIMERE cohort on Reunion Island

Raphaëlle Sarton [1] *, Magali Carbonnier [2], Stéphanie Robin [3,4], Duksha Ramful [5] , Sylvain Sampériz [5], Pascale Gauthier [6], Marc Bintner [6], Brahim Boumahni [7], Patrick Gérardin [8] *

[1] Department of Pediatrics, Centre Hospitalier Universitaire (CHU) de La Réunion, Saint Pierre, Reunion, France; raphaelle.sarton@chu-reunion.fr
[2] Centre d'Action Médico-Sociale Précoce Isautier, Fondation Père Favron, Saint Louis, Reunion, France; m-carbonnier@favron.org
[3] Department of Pediatrics, CHU de La Réunion, Saint Denis, Reunion, France
[4] Centre Ressources TSAF (Troubles du Spectre de l'Alcoolisation Fœtale), CHU de La Réunion, Saint Denis/Saint Pierre, Reunion, France; stephanie.robin@chu-reunion.fr
[5] Neonatal Intensive Care Unit, CHU de La Réunion, Saint Denis, Reunion, France; duksha.ramful@chu-reunion.fr & sylvain.samperiz@chu-reunion.fr
[6] Neuroradiology department, CHU de La Réunion, Saint Pierre, Reunion, France; marc.bintner@chu-reunion.fr
[7] Neonatalogy Unit, CHU de La Réunion, Saint Pierre, Reunion, France; brahim.boumahni@chu-reunion.fr
[8] Centre for Clinical Investigation Clinical Epidemiology (INSERM CIC 1410), CHU de La Réunion, Saint Pierre, Reunion, France; patrick.gerardin@chu-reunion.fr

* Correspondence: R.S. raphaelle.sarton@chu-reunion.fr ; P.Gé. patrick.gerardin@chu-reunion.fr

Table S1. Risk classification for cognitive and learning difficulties using the EDA (Assessment scale of child cognitive and learning functions- First edition) scales in a cohort of 21 children exposed to perinatal mother-to-child Chikungunya virus infection, Reunion island, 2015-2016

Risk	Abnormal/high risk (n=3)		Subnormal / at risk (n = 7)		Normal/low risk (n=9)	
**Functions**	*Z-score < - 2 SD*		*- 2 DS ≤ Z-score < - 1 DS*		*Z-score ≥ - 1 SD*	
Verbal (n = 19)	N	(%)				
Phonology	1	(5.3 %)	2	(10.5 %)	16	(84.2 %)
Lexical evocation	1	(5.3 %)	1	(5.3 %)	17	(89.5 %)
Lexical comprehension	1	(5.3 %)	0	(0.0 %)	18	(94.7 %)
Syntactic expression	2	(10.5 %)	1	(5.3 %)	16	(84.2 %)
Syntactic comprehension	1	(5.3 %)	1	(5.3 %)	17	(89.5 %)
Non-verbal (n = 18)						
Graphism	2	(11.1 %)	0	(0.0 %)	16	(88.9 %)
Visual selective attention	0	(0.0 %)	3	(16.7 %)	15	(83.3 %)
Planning	2	(11.1 %)	0	(0.0 %)	16	(88.9 %)
Visuospatial reasoning	1	(5.6 %)	1	(5.6 %)	16	(88.9 %)
**Learnings (n= 15)**						
Reading	1	(6.7 %)	3	(20.0 %)	11	(73.3 %)
Dictation	0	(0.0 %)	1	(6.7%)	14	(93.3%)
Mathematics	1	(6.7 %)	2	(13.3 %)	12	(80.0 %)

Data are numbers and row percentages. Children are classified according to their worst performance to 12 EDA subscales into three Z-score categories estimated from the study population. 2 children could not be assessed.

Table S2. Performances on EDA (Assessment scale of child cognitive and learning functions-First ed) scales in a cohort of 21 children exposed to perinatal mother-to-child chikungunya virus infection according to disease presentation, Reunion island, 2015-2016

**Disease group**	**Non – encephalopathic**			**Encephalopathic**			
**Functions**	n = 12			n = 7			
Verbal (n = 19)	Mean	SD	(95% CI)	Mean	SD	(95% CI)	*p value*
Phonology	19.3	1.2	18.6 - 20.1	12.7	7.6	5.7 - 19.7	**0.0407**
Lexical evocation	53.0	5.0	49.7 - 56.2	39.6	13.9	26.7 - 52.4	**0.0049**
Lexical comprehension	31.0	2.6	29.3 - 32.6	25.3	9.8	16.2 - 34.3	0.1469
Syntactic expression	18.7	2.3	17.2 - 20.2	11.9	7.7	4.7 - 19.0	**0.0023**
Syntactic comprehension	28.5	4.0	25.9 - 31.1	18.9	5.7	13.5 - 24.2	**0.0004**
Non-verbal (n = 18)							
Graphism	5.3	1.3	4.5 - 6.2	2.7	2.3	0.2 - 5.1	**0.0184**
Selective visual attention	20.1	5.3	16.7 - 23.5	14.0	4.5	9.2 - 18.7	**0.0257**
Planning	7.9	1.5	6.9 - 8.9	4.8	3.9	0.7 - 8.9	**0.0329**
Visuospatial reasoning	8.8	2.6	7.1 - 10.4	6.2	4.0	1.9 - 10.4	0.2179
**Learnings (n= 15)**							
Reading	27.6	9.0	21.8 - 33.3	13.0	18.4	-32.6 - 58.6	0.1846
Dictation	9.5	2.9	7.6 - 11.3	7.3	0.6	5.9 - 8.8	0.1934
Mathematics	10.4	2.9	8.6 - 12.2	5.5	4.3	-5.1 - 16.1	0.1143

Data are means, standard deviations (SD) and 95% confidence intervals (95% CI).

Means are compared using Mann-Whitney U tests. Significant p-values are in bold.

2 children could not be assessed.

Table S3. Performances on EDA (Assessment scale of child cognitive and learning functions-First ed) scales in a cohort of 21 children exposed to perinatal mother-to-child chikungunya virus infection, Reunion island, 2015-2016, and in a French national cohort of 626 healthy children

Disease group	Healthy controls			Non – encephalopathic			
**Functions**	n = 626			n = 12			
Verbal (n = 12)	Mean	SD	(95% CI)	Mean	SD	(95% CI)	*p value*
Phonology	19.8	0.6	19.7 - 19.8	19.3	1.2	18.6 - 20.1	0.1774
Lexical evocation	54.1	3.3	53.8 - 54.4	53.0	5.0	49.7 - 56.2	0.4635
Lexical comprehension	32.8	1.3	32.7 - 32.9	31.0	2.6	29.3 - 32.6	0.0355
Syntactic expression	17.6	1.5	17.4 - 17.7	18.7	2.3	17.2 - 20.2	**0.0131**
Syntactic comprehension	27.2	2.6	27.0 - 27.4	28.5	4.0	25.9 - 31.1	0.2856
Non-verbal (n = 12)							
Graphism	7.0	1.8	6.8 - 7.1	5.3	1.3	4.5 - 6.2	**0.0012**
Selective visual attention	23.4	4.7	23.0 - 23.8	20.1	5.3	16.7 - 23.5	**0.0165**
Planning	8.6	1.5	8.4 - 8.7	7.9	1.5	6.9 - 8.9	0.1098
Visuospatial reasoning	24.9	2.5	24.7 - 25.1	8.8	2.6	7.1 - 10.4	**<0.0001**
**Learnings (n= 12)**							
Reading	23.5	4.2	23.1 - 23.8	27.6	9.0	21.8 - 33.3	0.1433
Dictation	11.0	2.5	10.8 - 11.2	9.5	2.9	7.6 - 11.3	0.0405
Mathematics	16.5	2.6	16.3 - 16.7	10.4	2.9	8.6 - 12.2	**<0.0001**

Data are means, standard deviations (SD) and 95% confidence intervals (95% CI).

Means are compared using Student T tests. Statistical significance in bold was set at p=0.025 using a Bonferroni correction for accounting stratum level multiple comparisons to the same control group.

2 children could not be assessed.

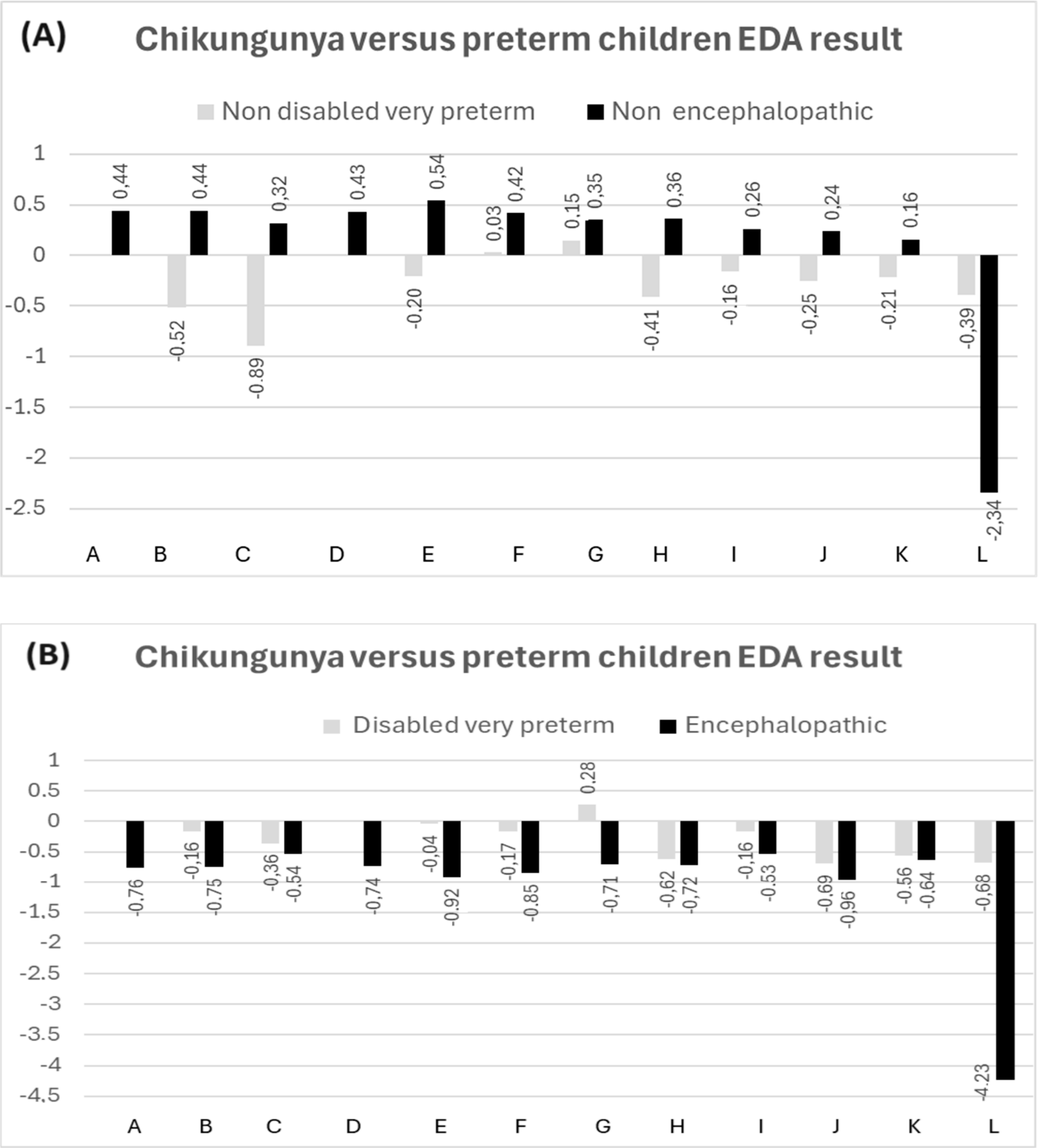

Figure S1. EDA Z-scores. (A) Mean Z-scores for each EDA subscale in CHIKV perinatally infected non-encephalopathic children and non-disabled very preterm infants from the Lorraine regional cohort. (B) Mean Z-scores for each EDA subscale in CHIKV perinatally infected encephalopathic children and disabled very preterm infants from the Lorraine regional cohort.

A: Phonology, B: Lexical evocation, C: Lexical comprehension, D: Syntactic expression, E: Syntactic comprehension, F: Graphism, G: Visual selective attention, H: Planning, I: Visuospatial reasoning, J: Reading, K: Dictation, L: Mathematics

Table S4. Performances on EDA (Assessment scale of child cognitive and learning functions- First ed.) scales in a cohort of 21 children exposed to perinatal mother-to-child Chikungunya virus infection according to maternal education level, Reunion island, 2015-2016

**Maternal education**	Elementary school		High school		Bachelor degree		College		
**Functions**	(n = 1)		(n = 9)		(n = 6)		(n = 3)		
Verbal (n = 19)	Mean	(Min – Max)	Mean	(Min – Max)	Mean	(Min – Max)	Mean	(Min – Max)	*P*-value
Phonology	0.02	(0.02 - 0.02)	0.32	(-1.42 - 0.56)	-0.10	(-1.78 - 0.56)	-0.76	(-3.05 - 0.38)	0.1259
Lexical evocation	0.26	(0.26 - 0.26)	0.25	(-0.54 - 0.89)	-0.39	(-3.23 - 0.89)	-0.06	(-1.44 - 0.80)	0.9865
Lexical comprehension	0.77	(0.77 - 0.77)	0.08	(-0.89 - 0.77)	-0.29	(-3.60 - 0.77)	0.07	(-0.73 - 0.62)	0.5569
Syntactic expression	-0.25	(-2.25 - -2.25)	0.15	(-1.05 - 0.64)	-0.04	(-2.75 - 0.64)	0.36	(-0.03 - 0.64)	0.5141
Syntactic comprehension	-0.90	(-0.90 - -0.90)	0.11	(-1.95 - 2.13)	-0.07	(-2.26 - 0.76)	0.11	(-0.74 - 0.61)	0.6265
Non-verbal (n = 18)									
Graphism	-0.69	(-0.69 - -0.69)	0.03	(-0.69 - 1.70)	-0.13	(-2.12 - 0.74)	-0.21	(-2.12 - 0.74)	0.7125
Selective visual attention	-1.06	(-1.06 - -1.06)	0.12	(-0.70 - 2.08)	-0.07	(-1.05 - 1.04)	0.16	(-1.75 - 1.21)	0.5349
Planning	0.39	(0.39 - 0.39)	0.43	(-0.65 - 0.73)	-0.37	(-2.40 - 0.74)	-0.54	(-2.40 - 0.39)	0.2690
Visuospatial reasoning	-0.27	(-0.27 - -0.27)	0.30	(-0.27 - 0.96)	-0.32	(-2.42 - 1.57)	-0.07	(-1.50 - 0.96)	0.7242
**Learnings (n= 15)**									
Reading	-2.02	(-2.02 - -2.02)	0.24	(-1.03 - 0.85)	-0.13	(-1.61 - 0.93)	0.50	(0.43 - 0.56)	0.4063
Dictation	-0.76	(-0.76 - -0.76)	0.13	(-0.76 - 2.56)	-0.32	(-1.50 - 0.71)	0.71	(0.34 - 1.08)	0.3576
Mathematics	-5.76	(-5.76 - -5.76)	-2.97	(-4.61 - -0.96)	-2.12	(-2.50 - -1.34)	-1.83	(-2.69 – 0.96)	0.1769

Data are Z-score means.

Means are compared using Kruskal-Wallis H tests.

2 children could not be assessed.

Table S5 (Excel file). Putative anatomical-clinical correlates between the observed cognitive dysfunctions and neuro-imagery findings in a cohort of 21 children perinatally-infected with chikungunya virus, Reunion island, 2015-2016

Patients who underwent both MRI scans and EDA evaluations are identified as a number.
